# Drivers of Irrational Use of Antibiotics in Europe

**DOI:** 10.3390/ijerph16010027

**Published:** 2018-12-23

**Authors:** Anna Machowska, Cecilia Stålsby Lundborg

**Affiliations:** Global Health-Health Systems and Policy: Medicines, Focusing Antibiotics, Department of Public Health Sciences, Karolinska Institutet, 171 77 Stockholm, Sweden; Cecilia.Stalsby.Lundborg@ki.se

**Keywords:** antibiotics, antibiotic resistance, irrational use, unnecessary antibiotic use, Europe

## Abstract

The unnecessary use of antibiotics and concomitant rapid growth of antibiotic resistance (ABR) is a widely acknowledged threat to global health, development, and sustainability. While the underlying cause of ABR is undoubtedly the overall volume of antibiotic use in general, irrational antibiotic use, which is influenced by several interrelated factors, is a major contributory factor. Here, we aimed to present and describe selected main drivers of irrational use of antibiotics in Europe. We performed a broad search of the current literature in databases such as PubMed, Google Scholar, Cochrane, as well as various institutional websites (World Health Organization, European Observatory, European Commission) to provide a new perspective on selected drivers of irrational antibiotic use in Europe. We also searched for relevant literature using snowballing, i.e., using reference lists of papers to identify additional papers. In this narrative review, we present that major factors among the general public driving antibiotic resistance are lack of public knowledge and awareness, access to antibiotics without prescription and leftover antibiotics, and knowledge attitude and perception of prescribers and dispensers, inadequate medical training, pharmaceutical promotion, lack of rapid and sufficient diagnostic tests, and patient–doctor interaction as major factors among healthcare providers. We further discuss initiatives that, if taken and implemented, can have an impact on and improve the current situation in Europe.

## 1. Introduction

### 1.1. Current Situation of Antibiotic Use and Resistance Worldwide and in Europe

Antibiotics are one of the most cost-effective, life-saving medicines and contribute to an extended lifespan [1]. However, the effect of antibiotics is compromised by the rapid escalation of antibiotic resistance (ABR), which, combined with the paucity of development of new antibiotics (or antibiotic combinations) with novel mode(s) of action is considered a major global health threat [2]. A key driver of ABR is the irrational use of antibiotics. According to the World Health Organization (WHO) definition, medicines are used rationally when patients receive the appropriate medicines, for appropriate indications, in doses that meet their own individual requirements, for an adequate period of time, at the lowest cost both to them and the society, and with appropriate information. Irrational or unnecessary use of medicines occurs when one or more of these conditions is not met [3].

Antibiotic resistance in common pathogens is consistently reported to be higher in countries that have a higher use of antibiotics [4]. The European Surveillance of Antimicrobial Consumption Network (ESAC-Net) showed that large variations in antibiotic use exist across Europe, with higher use in Southern Europe and lower in Northern Europe [5].

During the period 2012–2016, a statistically significant decreasing trend in antibiotic use was observed in Finland, Luxembourg, Norway, and Sweden, while an increasing trend was observed in Greece and Spain. In 2016, antibiotic consumption for systemic use in the community (i.e., outside hospitals) in Greece was 36.3 defined daily doses (DDD) per 1000 inhabitants per day (DDD/TID). Meanwhile, in the Netherlands, less than a third of this volume was used (10.4 DDD/TID).

Although only 10–20% of all antibiotics are used in hospitals, the intensity of use is far higher than in the community. Hospitals are key locations in which final recourse antibiotics—such as carbapenems and polmyxins—are used [5,6,7,8]. For example, ESAC-Net data showed a higher proportion of use of cephalosporins, other beta-lactams (including carbapenems), and other groups of antibiotics in hospitals compared to use in the community [5]. The proportion of carbapenem-resistant invasive *Klebsiella pneumoniae* is strongly associated with carbapenem consumption. Therefore, data showing that more than one million carbapenem prescriptions are issued in Europe each year is a major concern.

The health and economic consequences of ABR are severe. Today, drug-resistant infections lead to approximately 700,000 deaths per year globally. This is projected to increase to 10 million by 2050, with associated costs as high as US $100 trillion worldwide if no action is taken [6]. Each year, in the European Union (EU) alone, 25,000 patients die due to infections caused by multiresistant bacteria, costing society approximately €1.5 billion annually. By 2050, expected cumulative losses due to multiresistance will reach 2.9 trillion USD per year [7]. 

Although the absolute consumption of antibiotics is the largest in high-income countries, antibiotic consumption increases the fastest in low- and middle-income countries (LMICs) [9] which also face particular challenges in addressing antibiotic resistance [10]. However, it should be noted that the fast rise of antibiotic consumption started from low level of use and still, many people in LMICs suffer more from lack of access than excess use of antibiotics. Resistance spreads quickly across the globe and what is happening in LMICs, e.g., in terms of the spread of resistant bacteria and resistant genes has tremendous consequences for all European countries [11]. Therefore, strong international collaboration is needed to address this escalating situation promptly. 

### 1.2. Spread of Resistance Bacteria

Bacteria may become resistant to antibiotics via de novo gene mutation or by acquiring the genetic information that encodes resistance from other bacteria. The selective pressure imposed by the massive use of antibiotics makes bacteria carrying the resistance gene survive and grow [8]. A special concern is the rapid spread of multiresistant bacteria, reported in several countries, for some of which there is no available treatment. Data from the European Antimicrobial Resistance Surveillance Network (EARS-Net) showed substantial geographic differences in the proportion of resistance to various classes of antibiotics, with higher levels in Southern Europe, and lower in Northern Europe [4]. Although the combined resistance to fluoroquinolones, third-generation cephalosporins, and aminoglycosides of *Klebsiella pneumoniae* isolates overall did not increase in Europe between 2014 and 2017 (20.8% to 20.5%), increasing national trends were reported from several countries. In 2017, 7.2% of *Klebsiella pneumoniae* isolates were identified as resistant to carbapanems, with an enormous variation across Europe from 0% in several countries, such as Croatia, Estonia, Iceland, Luxembourg, Norway, and Slovenia, to 64.7% in Greece. At the same time, 14.9% of *Escherichia coli* isolates were identified as resistant to third-generation cephalosporins, ranging from 5.9% in Norway to 41.3% in Bulgaria. For *Escherichia coli* and *Klebsiella pneumoniae*, combined resistance to several antimicrobial groups was frequent, and extended-spectrum beta-lactamase (ESBL) production was common. A majority (87.4%) of the third-generation cephalosporin-resistant isolates from 2017 were extended-spectrum beta-lactamase (ESBL)-positive. Methicillin-resistant *Staphylococcus aureus* (MRSA) invasive isolates reached a proportion of 16.9%, with large variations across Europe (1.0% in Norway to 44.4% in Romania) [4].

**Figure 1:**
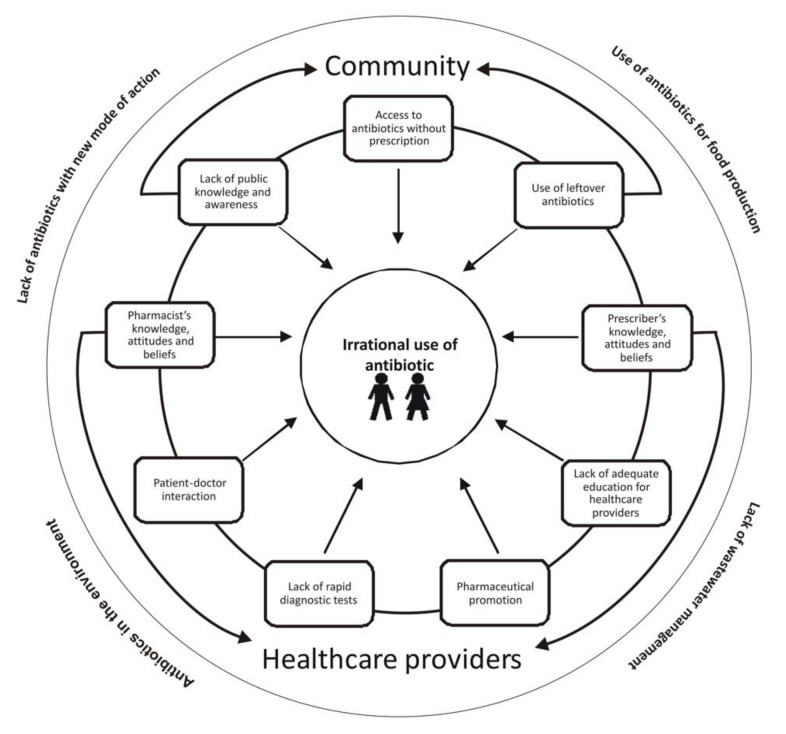
Examples of drivers or irrational antibiotic use in Europe.

To adequately address the threat of ABR, it is essential to understand the main factors driving irrational antibiotic use Europe. In this review, we describe main drivers of irrational antibiotic use among the general public and healthcare providers (HCPs) in Europe (see Figure 1). Additionally, other factors presented in Figure 1, such as paucity of development of new antibiotics (or antibiotic combinations) with novel mode(s) of action, lack of wastewater management, or One Health approach-related factors, such as antibiotics in the environment and use of antibiotics in the animal and food industry, were outside of the scope of this review, which focused only on human antibiotic use, and selective main drivers of non-responsible use. We also discuss effective actions urgently required from international organizations, governments, researchers, and private and public sector clinicians to minimise ABR through improved use of antibiotics.

### 1.3. Research Questions

We have posed several research questions to guide us through the literature search process. What is the current situation of antibiotic use and resistance in Europe? How did the situation change during recent years? What are the differences between European countries? What are the commonly discussed factors driving irrational antibiotic use in Europe in the community and among HCPs?

### 1.4. Literature Search

In order to respond to the research questions, we performed a broad search of the current literature using the following databases: PubMed, Google Scholar, Cochrane, as well as various institutional websites (World Health Organization, European Observatory, European Commission) to provide a new perspective on broadly discussed drivers of irrational antibiotic use in Europe. The following terms were used as phrases to search the databases in a way suitable for the specific database: Antibiotic use, antibiotic consumption, antibiotic usage, antibiotic utilization, anti-bacterial agents, antibiotic *, antibacterial *, antimicrobial *, anti-microbial *, prescribing, prescription *, dispensing, dispensation *, inappropriate prescribing, overprescribing, overprescription, overuse, pattern *, medicine use, prudent, drug misuse, medicine misuse. We also searched for relevant literature using snowballing, i.e., using reference lists of papers to identify additional papers. We also selected key reports from international organizations to serve as reference European data. The literature search elicited the leading themes and the main papers within the themes were scrutinised, compared, and contrasted. The recurring issues within the published papers are presented and discussed.

## 2. Drivers of Irrational Use of Antibiotics among the General Public

### 2.1. Lack of Public Knowledge and Awareness

Public knowledge, attitudes, and beliefs about antibiotics are strong determinants of irrational use of antibiotics. The latest Eurobarometer report showed that 34% of Europeans took antibiotics at least once during 2016 [12]. There is a clear need to raise awareness about antibiotic use and resistance among European populations. For example, 57% are unaware that antibiotics are ineffective against viruses, and 44% do not know that antibiotics have no effect against cold and influenza. There are large differences between countries and social groups. The use of antibiotics is shown to be higher among those with a lower level of education (39% compared with 32–33%) and those in worse economic circumstances (44% compared with 31%) [12]. The opposite, however, was shown in a study from Southern Sweden, where socioeconomic factors reflecting a privileged situation correlated positively with higher antibiotic use, especially among children aged 0–6 years [13]. In districts with a high median family income and a high employment rate, the use of antibiotics was higher than in other districts. That suggests that antibiotic use among children may, in some cases, increase with the degree of parental affluence but cannot be solely explained by economic factors. 

People often have an incomplete understanding of—and misconceptions about—ABR. Many believe that they themselves do not contribute to the development of ABR and do not understand that bacteria—and not humans—become resistant [14]. A comparative European study found the highest level of misconception contributing to inappropriate use in Southern and Eastern Europe. The highest prevalence of ABR is found in countries where people have the lowest awareness about the issue. The underlying factor here is cultural differences in public attitudes, beliefs, and knowledge about antibiotic use, resistance, and self-medication [15]. A Lithuanian study showed that two-thirds of participants had an insufficient level of knowledge about antibiotics, and that participants tended to overestimate their knowledge, which may lead to increased non-adherence and self-medication [16]. According to the Eurobarometer, sources of information about antibiotics vary across countries, but overall, only 32% of Europeans stated that they received information about correct use of antibiotics from doctors. When asked where, in the last 12 months, they had received information on the correct use of antibiotics, 10% of Europeans said it came from pharmacists, 27% from TV advertisements, 26% from TV news, 19% from newspapers, and 13% looked for information online [12].

### 2.2. Access to Antibiotics without Prescription

Access to antibiotics without a prescription is a driving factor for irrational antibiotic use due to a potential lack of access to proper diagnosis and diagnostic tools. This eventually leads to the development and spread of ABR. Ninety-three percent of Europeans stated that they obtained antibiotics on prescription or directly from a medical practitioner. However, despite the legal framework stating that antibiotics should only be dispensed with a medical prescription and that over the counter (OTC) sales of antibiotics are illegal in all Member States of the European Union (there are some exceptions in a number of them—for example, creams or eye drops that contain antibiotics), 4% of Europeans reported obtaining their last course of antibiotics without a prescription [12]. In Greece, only 79% of respondent said they received antibiotics on prescription from a medical practitioner, whereas, in Sweden, that figure was 98% [12]. A survey from Algarve region in Portugal showed that of 1198 respondents, 7.5% answered that it was easy to buy antibiotics without a prescription [17]. The dispensing process greatly influences how antibiotics are used. In some countries, it is easier to buy antibiotics in community pharmacies without a prescription from a physician. A study from Catalonia, Spain (2009) showed that antibiotics were sold without a prescription in 55 out of 69 (79.7%) of studied pharmacies, in which a simulated case of a urinary tract infection (UTI) was presented [18]. It must, however, be acknowledged that European countries have different approaches towards the treatment of uncomplicated UTIs. In the UK, UTIs are one of the most common acute medical conditions, accounting for 1–3% of all general practitioner (GPs) consultations a year. To improve patient access to treatment—and decrease the number of consultations—in one area in Scotland, pharmacists can now offer antibiotics to treat uncomplicated UTIs (https://www.chemistanddruggist.co.uk/news/pharmacists-gain-key-role-new-scottish-antibiotics-scheme).

To facilitate easier access to medicines, in some EU Member States, medicines can be authorised for sale or supply as an OTC if it is safe to do so. The antibiotics available OTC are usually dispensed under the supervision of a pharmacist. A survey in 26 European countries and Norway identified 48 antibiotic formulations, containing 20 different active substances available OTC. Most of these products are used mainly as topical preparations or eye drops in short treatment courses. Although this should not be confused with the illegal supply of antibiotics that require a prescription, the spread of ABR makes it important to limit the availability of licensed OTC antibiotics and to monitor their use [19].

The use of antibiotics without a prescription, obtained via the internet or bought in another country, is considered to be a growing problem, as reflected in a European survey [20]. There are numerous international online pharmacies operating illegally outside the EU that can supply European patients by post or courier. These online vendors are neither authorised to operate in the EU nor do they adhere to national practices and guidelines—for example, they offer antibiotics for sale without a prescription. To prevent this, all online pharmacies in EU are currently required to display a logo, which acts as a direct link, enabling the checking of the legal status of the pharmacy via the Member State’s official pharmacy regulator [21]. 

Another issue is the increasing access to internet doctors (in Sweden, for example, called Kry doctor, https://kry.se/en/) who provide online consultations via a video call and may prescribe antibiotics legally, without any medical examination. Although there are no published evidence of irrational antibiotic prescribing resulting from those consultations, it might be a possible source of it. It is important to bear in mind that it is the lack of a proper examination and potential testing before the antibiotic is prescribed that leads to irrational antibiotic use. 

### 2.3. Leftover Antibiotics

Leftover (remaining) antibiotics from earlier prescriptions, when the patient did not adhere to the therapy, or the quantity of prescribed antibiotics exceeded the treatment duration, facilitate the practice of self-medication [22]. According to the Eurobarometer, 2% of Europeans use antibiotics leftover from previous courses [12]. Data from a UK survey of 6983 households showed that 19% of those surveyed had a leftover drug. Prescriptions for >6 days constituted 61% of leftover drugs, whereas prescriptions for <3 days constituted 6% of leftover drugs [22]. Evidence shows that prolonged or repeated treatment with antibiotics provides a greater selective pressure on normal bacterial flora than a single course of treatment, favouring the emergence of resistant strains [23,24].

## 3. Drivers of Irrational Use of Antibiotics among Healthcare Providers

### 3.1. Knowledge, Attitude, and Perception of Healthcare Providers Regarding Antibiotic Use and Resistance

Prescribers are ultimately responsible for making the decision to use antibiotics and for the selection of the type of antibiotic. Their knowledge, attitudes, and how they perceive antibiotic use and resistance is likely to influence prescribing behaviour. It has been suggested that the attitude and knowledge of physicians determines the quality of prescribing of antibiotics, as measured by indicators obtained from clinical practice. The results of a Spanish study indicated that fear of complications from infections, a complacent attitude towards patients, and insufficient knowledge regarding ABR are factors related to the prescribing of antibiotics by general practitioners [25]. Data from Sweden, however, show that the reduced rate of antibiotic prescriptions in Sweden have not increased complications of the infections due to not treating with antibiotics, e.g., the incidence of mastoiditis seems not to increase in children despite a reduction in antibiotic prescriptions for otitis media [26].

In a recent systematic review, it was shown that physicians generally believed that ABR was a serious problem but not in their close proximity. The number of clinicians who believed it was a problem at the local, national, or global level was greater than the number who believed it was a problem at their practice level [27]. A qualitative study from Sweden showed that some GPs did not consider resistance as a problem they experienced in their everyday practice. Although some GPs were aware of the problem, this had a minor impact on their own practice, considering it as a problem found in other countries, other parts of the country, or in hospitals. GPs’ perceptions of ABR were mirrored in how they reported their treatment of UTIs in practice [28]. In addition to attitudes, the workload and working at the emergency departments have been identified as critical factors affecting antibiotic prescribing [29].

### 3.2. Lack of Adequate Education for Healthcare Providers 

Inadequate training on antibiotic prescribing during medical education and further on during the early stage of clinical practice, as well as inadequate continuing professional development throughout professional careers, can contribute to inappropriate antibiotic prescribing. The WHO recently highlighted the importance of undergraduate training in prudent prescribing behaviour [26]. A recent survey on self-reported preparedness among final-year medical students in 29 European countries showed that on average, 66.1% of students (ranging from 20.3% in Sweden to 94.3% in Slovakia) wanted more education on prudent antibiotic use or general antibiotic use [30]. A survey of 140 junior doctors at 5 London hospitals found that only 5–13% of participants thought their previous education on the use of antibiotics was sufficient. Sixty percent of those in their first-year post-qualification reported wanting further education on antibiotic prescribing, rising to 74% among more experienced junior doctors [31].

### 3.3. Pharmaceutical Promotion

Pharmaceutical promotion is also believed to increase irrational prescribing. The EU regulation on pharmaceutical promotion states that advertising must be consistent with the approved product information and cannot be misleading. It also states that Member States shall ensure that there are adequate and effective methods to monitor the advertising of medicinal products. However, it has been argued that, globally, even countries with adequate resources for regulatory oversight vary greatly in the extent to which they effectively monitor pharmaceutical promotion and enforce laws with adequate sanctions [32]. Pharmaceutical promotion influences how healthcare professionals prescribe and dispense. Many studies included in a systematic review published in 2010 showed that physicians’ exposure to information from pharmaceutical companies is associated with higher prescribing frequency, higher costs, and lower prescribing quality. However, the heterogeneity of the included studies must be acknowledged [33]. An effective technique for developing relationships that influence prescribing behaviour is one-to-one contact between doctors and sales representatives. In fact, sales representatives are highly trained in persuasion and influencing skills. A recent study from Germany showed that doctors who frequently saw pharmaceutical sales representatives had a higher number of total prescriptions compared to doctors who were visited less frequently [34]. In a qualitative study from Spain, focus group discussions among 33 physicians explored their habits and knowledge with regard to antibiotics and aimed to identify factors that may influence prescribing. It was shown that physicians attributed a very clear influence to pharmaceutical promotion and advertising when it came to choosing antibiotics. Some of the participants stated:

‘The influence of the pharmaceutical industry is so clear that, when they stop promoting a medication, then, in the long term, you too stop using it’.

Or,

‘We are constantly being bombarded by the pharmaceutical industry because they keep on saying that this is the latest cephalosporin, the best, the one that’s recommended in all the guidelines for the treatment of increased expectoration in COPD, and it’s a lie; and so that’s what we have to fight against, […]’.

[35]

### 3.4. Lack of Rapid and Sufficient Diagnostic Tests and Local Antibiotic Susceptibility Data

A core problem behind the incorrect prescribing and use of antibiotics is the lack of sufficient diagnostic tests to rapidly identify the pathogen and its antibiotic susceptibility profile, guide antibiotic prescribing at the point of care, and reduce the need for broad spectrum antibiotics [36]. Conventional culturing and susceptibility testing takes time; thus, in most cases, empiric prescribing always precedes culture results. In all countries, empiric prescribing dominates, but the choice of antibiotics might be changed to narrower spectrum ones a culture is done and after-culture results are available. New approaches in diagnostic technologies, such as nucleic acid amplification tests (NAAT), matrix-assisted laser desorption ionization-time of flight mass spectrometry (MALDI-TOF), and antigen detection, have enabled timely antibiotic optimization [37,38]. However, the availability of the rapid diagnostics does not necessarily result in improved antibiotic prescribing. It was shown that the concomitant presence of an antibiotic stewardship programme is crucial and results in a more rapid improvement of antibiotic prescribing [39]. Moreover, the important limitation of rapid diagnostic tests is their low performance on antibiotic susceptibility testing. Although some methods can identify certain resistance markers, these tests are still incapable of detecting full antibiotic susceptibility profiles of pathogens. The selection of a suitable diagnostic test should be contextualised, adjusted to local prescribing guidelines and the prevalence of pathogens, and integrated with laboratory workflow.

The availability of regional antibiotic susceptibility data for most common bacteria to inform medical practitioners vary across European countries. Many countries report the data only from a small number of hospitals in a specific geographical location. Therefore, the sample cannot be representative and may not show variations at the regional level within a country [4]. The sample of patients included in surveillance should aim to consist of a mix of patient types (e.g., paediatric, ICU, or neurosurgery patients) and infection types (e.g., community-acquired urosepsis and healthcare-associated bloodstream infections), in proportion to their occurrence in the total population.

In 2015, the WHO launched the Global Antimicrobial Resistance Surveillance System (GLASS) [40], the first global collaborative effort to standardise antimicrobial resistance surveillance. GLASS provides a standardised approach to the collection, analysis, and sharing of antibiotic resistance data by countries all over the world and seeks to document the status of existing or newly developed national surveillance systems.

### 3.5. Patient–Doctor Interaction

Many studies have shown the relevance of the patient-doctor relationship and communication for the prescribing of antibiotics. A qualitative study from UK showed that doctors overestimated patients’ expectations for antibiotics and prescribed antibiotics to maintain a good patient-doctor relationship [41]. A survey of 1000 GPs in the UK showed that 55% felt under pressure to prescribe antibiotics, even if they were not sure that they were necessary, and 44% admitted that they had prescribed antibiotics to get a patient to leave the surgery [42].

The consequences of antibiotic misprescribing and/or overprescribing are severe and have a major impact on the development of ABR, the medicalisation of patients’ symptoms, and increasing healthcare costs [43]. The vast majority of antibiotic prescriptions for systemic use in primary care, for both adults and children, is for respiratory tract infections (RTIs) [44]. The rate of unnecessary prescribing for RTIs, for which antibiotics are rarely indicated, ranges from one-half to 90% [45,46,47]. A study from Poland showed overuse of antibiotics in primary care, where 50% of patients with a common cold received antibiotics [48]. Moreover, RTIs are most commonly self-limited and normally, no antibiotic course is needed. The expected time course of symptoms of RTIs last on average from one week for an acute sore throat, one and a half weeks for a common cold, and three weeks for an acute cough or bronchitis, which is comparable to a standard antibiotic course [49]. 

### 3.6. Knowledge, Attitude, and Perception of Pharmacists Regarding Antibiotic Use and Resistance

As part of the standard dispensing process, pharmacists in community and hospital settings provide counselling (advice, information, and help) on the safe, effective, and rational use of medicines [50]. A study conducted by the WHO showed that pharmacists are among the best-positioned healthcare professional group to tackle ABR [51]. Pharmacies are often the first point of contact with the healthcare system and are important in advising patients on symptomatic self-care without antibiotics or referring them to medical professionals for examination.

In some countries, dispensing without a prescription is still a common practice. A study from Spain showed that pharmacists attributed the problem of antibiotics dispensed without a prescription—and its relationship to ABR—to the attitudes and/or factors, such as the external responsibility of doctors, dentists, and the National Health Service, acquiescence, and lack of continuing education [52]. A study from Portugal among community pharmacists showed that pharmacist knowledge and attitudes could influence their propensity to dispense antibiotics without earlier medical prescription. The attitudes most significantly influencing the propensity to dispense antibiotics without prescription were complacency about patients, responsibilities of others, and fear or precaution. Pharmacists had the highest degree of agreement to dispense without a medical prescription in cases of dental diseases and ailments (38.4%) and urinary tract infections (36.2%) [53].

A study from Romania evaluating the perceptions and attitudes of pharmacists toward their role in respect to antibiotic consumption and resistance showed that pharmacists encounter many barriers in their activities related to antibiotic management, such as when they see how the healthcare system impacts the patient’s ability to seek care [54]. The financial situation of a patient plays a major role in antibiotic consumption; this in turn impacts the role of the pharmacist. Combined with structural barriers created by the law, this can lead to ethical dilemmas about antibiotic management. Pharmacists knowing patients’ financial situation and their obstacles in accessing care may prioritise patients’ health outcomes and dispense antibiotics without prescription. 

## 4. Proposed Plans for Action

To minimise further increase in ABR, we hereby propose immediate and effective actions needed to improve use of antibiotics, from all stakeholders, including international organizations, governments, researchers, and private and public sector clinicians. 

### 4.1. Increase Public and Healthcare Professional Awareness about Antibiotic Resistance and Rational Use 

Providing education at all levels (community, healthcare, and individual) is essential to ensure rational use of antibiotics and to suppress the spread of ABR. Public education campaigns have been shown to be effective in changing attitudes and knowledge regarding antibiotic use and resistance [55,56] and should be further targeted at Southern and Eastern European countries reporting the highest levels of resistance. Since 2008, the European Centre for Disease Prevention and Control (ECDC) has coordinated “European Antibiotic Awareness Day”—an initiative that provides a platform for, and support of, national campaigns to raise awareness about the prudent use of antibiotics.

Improving education for healthcare professionals has recently been widely promoted as a potential method for optimising antibiotic use [44]. Special emphasis should be put on antibiotic prescribing training for medical students and junior doctors. Additionally, education on pharmaceutical promotion (including strategies used by companies, how they impact prescribing and dispensing, and practicing evidence-based medicine) should be adequately embedded in the curricula of medicine and pharmacy training. Furthermore, joint, multifaceted intervention targeting physicians, pharmacists, and the general public, which has been shown to effectively reduce the overall antibiotic use, should be considered [57].

Clinicians should support the education of patients regarding antibiotic use and resistance. For example, they could use effective strategies, such as shared decision making, to alert people to the actual risk of acquiring antibiotic-resistant bacteria following antibiotic use. Patients need to stay informed and receive independent information on antibiotics, as better health literacy and a higher degree of knowledge and awareness about the appropriate use of antibiotics are associated with decreased consumption. 

### 4.2. Optimise Antibiotic Use

Preventing leftovers from previous courses of antibiotics may be one effective way to prevent self-medication with antibiotics. This can be done through technical measures, such as promoting the dispensing of the exact number of tablets, or by educating patients not to use leftover medications. Moreover, take-back programmes, including the return of unused or excess drugs to pharmacies, are recommended by the WHO [58]. Most people in Europe are able to return expired or unused medicines to their community pharmacy—encouraging this is crucial to preventing the inappropriate use of antibiotics [21]. Infection prevention and control measures, included as one of the five objectives in the WHO Global Action Plan on Antimicrobial Resistance, are also crucial in order to reduce the incidence of infections and, therefore, the potential use of antibiotics. 

### 4.3. Strengthen the Data about Antibiotic Use and Resistance

Surveillance is one of the cornerstones of combating ABR. Lack of surveillance can lead to misdirected and inefficient policies and waste of resources. It is essential to understand trends in antibiotic use and resistance, inform treatment guidelines, identify priorities for intervention, tailor and target interventions, and monitor the impact of interventions. In a very recent draft opinion on ABR, developed by the European Parliament, it was emphasised that routine collection and submission of monitoring data at the EU level should be mandatory [59]. 

### 4.4. Mapping Information Regarding Antibiotics Use and Resistance

The WHO has recently undertaken many initiatives to map information regarding antibiotic use and resistance. The implementation status of countries’ action plans to address ABR across all sectors can be monitored via the WHO website [60]. In Europe, networks such as ESAC-Net and EARS-Net have been providing information regarding antibiotic use and resistance for over 15 years. Recently, the Access to Medicine Foundation published the methodology for its 2018 Antimicrobial Resistance Benchmark. It is the first independently developed framework for assessing how pharmaceutical companies are taking action to limit ABR. Activities by 30 companies are being mapped to help to identify obstacles and opportunities to tackle ABR [61]. The first Antimicrobial Resistance Benchmark report showed that out of 28 antibiotics in the late stage of clinical development, only 2 have both access and stewardship plans in place, nearly half of companies are involved in ABR surveillance, and 8 are setting limits on antibiotic wastewater discharge.

### 4.5. Support Public Health Driven Models of Innovation for the Development of Antibiotics

There is a shortage of a coordinated priority setting in antibiotic research and development spending. The current pipeline demonstrates a lack of truly valuable new antibiotics, vaccines, and diagnostics. Moreover, most publicly funded research and development initiatives in this area target the basic research phase. Much less funding is available for later stages of antibiotic development, clinical studies, and post-marketing surveillance, which require continuous financial support. 

Focused research is needed to help to develop new, effective treatment principles. The pharmaceutical industry has an important role in the overall effort to ensure the just and prudent use of antibiotics. Product development partnerships (PDPs) have the potential to address the lack of development of new antibiotics and to focus on unmet medical needs and should always include stewardship principles of any treatments that result from collaboration.

Innovative, affordable, and easy-to-use rapid diagnostic tests are needed to allow immediate identification of potentially infective organisms and to support doctors in deciding whether or not to initiate antibiotic therapy and thus improve the management of infectious diseases. It is also crucial to identify novel therapies that do not drive resistance. Numerous monoclonal antibodies have shown the potential to treat infectious diseases in preclinical evaluation, both in vitro and in animal models. As of November 2016, there were at least 38 monoclonal antibody products in active clinical development for 14 infectious diseases [62].

Research and development incentives based on the principle of delinkage should be applied in lieu of the traditional reward of market exclusivity (such as patents) to ensure the affordability of end products. The WHO and Drugs for Neglected Diseases initiative project on the Global Antibiotic Research and Development Partnership (GARDP) is a good example of an alternative, public health-driven model of innovation. More stewardship from governments regarding research and development agendas is needed. 

In the EU-supported Driving Reinvestment in Research and Development for Antibiotics and Advocating their Responsible Use (DRIVE-AB) project conducted from October 2014 to December 2017, several incentives have been determined to be the most effective in stimulating the antibiotic pipeline and ensuring that critical antibiotics continue to be accessible and can be used sustainably [63]. Grants given to academic or private institutions, pipeline coordinators that closely track the antibiotic pipeline and identify research and development gaps, market entry rewards, and long-term supply continuity models are complementary incentives which, if applied together, may ensure sustainable use and access. However, those findings remain without a follow-up in the European arena. By contrast, prompt actions are being taken in, for example, the US, where the Biomedical Advanced Research and Development Authority (BARDA) [64] and the Generating Antibiotic Incentives Now (GAIN) Act contributed to enhanced antibiotic development and more than doubled the yearly approval rate from 2000–2012 (0.8 approvals per year) to 2013–present (1.8 approvals per year) [65]. 

### 4.6. Stronger Regulations Governing Pharmaceutical Promotion

Governments should ensure proactive and effective control of pharmaceutical promotion, taking into account the threat to public health of antibiotic overuse and misuse. At a minimum, all promotional material on antibiotics should undergo a system of pre-vetting. Pharmaceutical products with antibiotic components should only be available as prescription only (PO), except where the healthcare system requires otherwise. 

### 4.7. Policy Coherence and Adequate Implementation

Political will and coherence at the European level is needed to achieve an ambitious plan to reduce the emerging growth of ABR.

The EU has taken several actions to mitigate the growing problem of ABR. After a revision of its Action Plan against the rising threats from antimicrobial resistance, implemented during 2011–2016, on 29 June 2017, the EU launched a new, more comprehensive One Health Action Plan. It contains 75 actions aimed at supporting the EU and its Member States in delivering effective and sustainable responses to ABR. The new EU One Health Action Plan takes a multisectoral approach, links EU and international actors, and mentions the possibility for funding Member States’ actions against ABR and boosting research and innovation. However, a number of shortcomings, such as the lack of a precise description of actions to support Member States and the engagement of various stakeholders that threaten this ambitious EU goal, have been identified. It was acknowledged that in order to achieve the common goal of reducing ABR, support for the development and implementation of national action plans and the allocation of adequate funds must be provided to the countries with the highest prevalence of ABR—and probably the lowest level of resources (human and financial)—and that strong national organisational structures are essential. The successful adoption and implementation of the Action Plan depends on the input of various actors—such as NGOs—who should be involved. The available and newly generated data should be used through the Action Plan as a benchmark for national reduction targets [66]. 

## 5. Conclusions

The severe health and economic consequences of ABR are well-recognised. The described drivers of irrational antibiotic use show the complexity of the problem that needs to be tackled from different angles. Although the number of initiatives and actors working on ABR is massive, they all emphasise the importance of urgent action to combat ABR, the need for international, cross-sectorial collaboration, education of the public and healthcare professionals, and foremost, the importance of a One Health approach. Humans, animals, the food chain, the environment—and the interconnectedness among them—should be treated as one entity to enhance public and animal health, and to benefit European and national economies.
